# Structural insights into human Arginase-1 pH dependence and its inhibition by the small molecule inhibitor CB-1158

**DOI:** 10.1016/j.yjsbx.2019.100014

**Published:** 2019-11-26

**Authors:** Yvonne Grobben, Joost C.M. Uitdehaag, Nicole Willemsen-Seegers, Werner W.A. Tabak, Jos de Man, Rogier C. Buijsman, Guido J.R. Zaman

**Affiliations:** Netherlands Translational Research Center B.V., Kloosterstraat 9, 5349 AB Oss, The Netherlands

**Keywords:** ABH, (2*S*)-2-amino-6-boronohexanoic acid, nor-NOHA, *N*^ω^-hydroxy-nor-L-arginine, IC_50_, half-maximal inhibitory concentration, *K*_D_, binding affinity, *K*_i_, inhibition constant, SPR, surface plasmon resonance, ITC, isothermal titration calorimetry, *K*_M_, Michaelis constant, *k*_cat_, catalytic rate constant, *T*_m_, melting temperature, Δ*T*_m_, melting temperature shift, SD, standard deviation, *k*_a_, association rate constant, *k*_d_, dissociation rate constant, τ, target residence time, RMSD, root-mean-square deviation, PDB, Protein Data Bank, MQ, MilliQ water, DMSO, dimethyl sulfoxide, Cancer immunotherapy, Biochemical inhibition, Surface plasmon resonance, Thermal stability, X-ray crystallography

## Abstract

Arginase-1 is a manganese-dependent metalloenzyme that catalyzes the hydrolysis of L-arginine into L-ornithine and urea. Arginase-1 is abundantly expressed by tumor-infiltrating myeloid cells that promote tumor immunosuppression, which is relieved by inhibition of Arginase-1. We have characterized the potencies of the Arginase-1 reference inhibitors (2*S*)-2-amino-6-boronohexanoic acid (ABH) and *N*^ω^-hydroxy-nor-L-arginine (nor-NOHA), and studied their pH-dependence and binding kinetics. To gain a better understanding of the structural changes underlying the high pH optimum of Arginase-1 and its pH-dependent inhibition, we determined the crystal structure of the human Arginase-1/ABH complex at pH 7.0 and 9.0. These structures revealed that at increased pH, the manganese cluster assumes a more symmetrical coordination structure, which presumably contributes to its increase in catalytic activity. Furthermore, we show that binding of ABH involves the presence of a sodium ion close to the manganese cluster. We also studied the investigational new drug CB-1158 (INCB001158). This inhibitor has a low-nanomolar potency at pH 7.4 and increases the thermal stability of Arginase-1 more than ABH and nor-NOHA. Moreover, CB-1158 displays slow association and dissociation kinetics at both pH 9.5 and 7.4, as indicated by surface plasmon resonance. The potent character of CB-1158 is presumably due to its increased rigidity compared to ABH as well as the formation of an additional hydrogen-bond network as observed by resolution of the Arginase-1/CB-1158 crystal structure.

## Highlights

• Side-by-side biochemical comparison of the inhibitors ABH, nor-NOHA and CB-1158.
• Arginase-1 binding, inhibition and stabilization by ABH and CB-1158 is pH-dependent.
• ABH and CB-1158 have slow association kinetics and a long target residence time.
• At higher pH, the catalytic center adopts a more symmetrical coordination structure.
• CB-1158 forms an additional hydrogen-bond network in the active site compared to ABH.

## Graphical abstract

**Figure:**
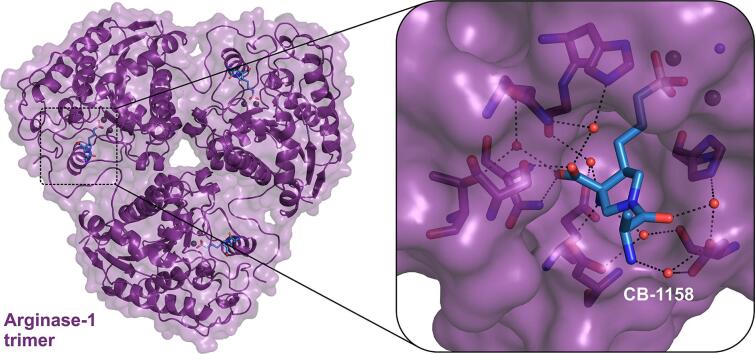

## 1 Introduction

The ability of tumors to modify their microenvironment and thereby evade the immune system of the host is increasingly recognized as an important determinant of cancer progression and patient prognosis (Gooden et al., 2011, Galon et al., 2013). The development of immune checkpoint therapies is an effective strategy to enhance anti-tumor immune responses of T-cells, for example using antibodies against cytotoxic T-lymphocyte-associated protein 4 (CTLA-4) (Hodi et al., 2010) or programmed cell-death 1 (PD-1) (Robert et al., 2015). However, the clinical response of these therapies is often limited by various resistance mechanisms (Jenkins et al., 2018), such as immunosuppression induced by the tumor myeloid compartment. One of the most prominent mechanisms contributing to this immunosuppression is the expression of Arginase-1 in the tumor microenvironment (Gabrilovich et al., 2012, Munder, 2009).

Arginase-1 (L-arginine amidinohydrolase, EC 3.5.3.1) is a manganese-dependent enzyme responsible for the catalytic hydrolysis of L-arginine into L-ornithine and urea. In the tumor microenvironment, enhanced expression of Arginase-1 by myeloid cells causes the local depletion of the semi-essential amino acid L-arginine. This results in anergy of effector T-cells by inhibition of CD3ζ chain expression (Rodriguez et al., 2004), and induces the suppression of effector T-cell and natural killer cell proliferation (Steggerda et al., 2017, Oberlies et al., 2009). Reduced levels of intracellular L-arginine can also directly impact the survival of activated T-cells (Geiger et al., 2016). Elevated levels of Arginase-1 have been detected in tumors of patients with various types of cancer, with the highest levels in lung, gastrointestinal and bladder cancers (Steggerda et al., 2017). Furthermore, elevated plasma Arginase-1 and reduced L-arginine levels are correlated with suppressed T-cell function and proliferation in patients with different histologies (Zea et al., 2005, Czystowska-Kuzmicz et al., 2019). Pharmacological inhibition of Arginase-1 has been shown to increase tumor immune cell infiltration and reduce tumor growth in syngeneic mouse models (Rodriguez et al., 2004, Steggerda et al., 2017, Czystowska-Kuzmicz et al., 2019, Narita et al., 2013, Miret et al., 2019). Arginase-1 is therefore an attractive target for the development of new drugs for cancer immunotherapy.

**Fig. 1:**
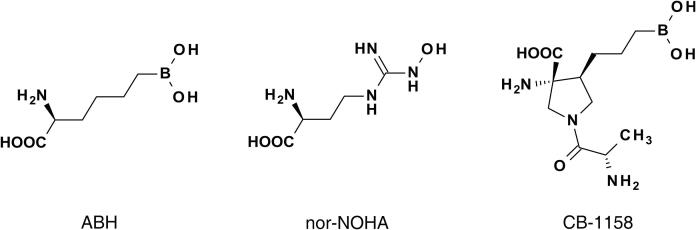
Chemical structures of the Arginase-1 inhibitors characterized in this study.

Aside from immuno-oncology, Arginase-1 has been a drug target for several decades for a variety of diseases and disorders, including pulmonary and vascular disease, and erectile dysfunction (Caldwell et al., 2018). Some broadly studied Arginase-1 inhibitors in this context are the boronic acid derivative (2*S*)-2-amino-6-boronohexanoic acid (ABH) (Baggio et al., 1997) and the L-arginine analogue *N*^ω^-hydroxy-nor-L-arginine (nor-NOHA) (Fig. 1) (Custot et al., 1997). The clinical application of both types of inhibitors is however limited by poor pharmacokinetic properties (Golebiowski et al., 2013, Havlinova et al., 2013). In addition, the boronic acid functionality of ABH may display cross-reactivity towards other proteins, causing potential toxicity (Ivanenkov and Chufarova, 2014). Recently, efforts aiming at the improvement of ABH for cancer immunotherapy have resulted in the development of the Arginase-1 inhibitor CB-1158 (INCB001158) by Calithera Biosciences, Inc. (South San Francisco, CA) (Steggerda et al., 2017, Golebiowski et al., 2013, Van Zandt et al., 2013, Golebiowski et al., 2013). CB-1158 is an orally bioavailable inhibitor, which reportedly inhibits human Arginase-1 in a biochemical assay with a half-maximal inhibitory concentration (IC_50_) of 86 nM (Steggerda et al., 2017). CB-1158 is currently evaluated for the treatment of advanced and metastatic solid tumors as a single agent, and in combination with chemotherapy, immune checkpoint therapy, and the IDO1 inhibitor epacadostat (see for example www.clinicaltrials.gov under NCT02903914, NCT03314935, and NCT03361228).

Biochemical inhibition of human Arginase-1 (and the isozyme Arginase-2) is reported in multiple studies, as summarized for ABH, nor-NOHA and CB-1158 in Table 1. Remarkably, there is significant discrepancy between the reported (low-)nanomolar binding affinities (*K*_D_) and the low-micromolar IC_50_ values of ABH and nor-NOHA (Table 1). Moreover, inhibition of Arginase-2 by ABH is reported to be pH-dependent, having an inhibition constant (*K*_i_) of 0.25 µM at pH 7.5 and a *K*_i_ of 8.5 nM at pH 9.5 (Colleluori and Ash, 2001). However, it is unclear if the same holds for Arginase-1. It is worth noting that inhibitory potencies reported in the literature for human Arginase-1 (and Arginase-2) are generally determined at physiological pH 7.4 (Steggerda et al., 2017, Van Zandt et al., 2013, Golebiowski et al., 2013, Van Zandt et al., 2019), whereas binding affinities were measured at pH 8.5 (Di Costanzo et al., 2005, Ilies et al., 2011, Di Costanzo et al., 2010). However, the pH optimum of human Arginase-1 lies between pH 9.0 and 10.0 (Zakalskiy et al., 2012, Jenkinson et al., 1996, Cabello et al., 1961, Berüter et al., 1978).

**Table 1:** Potencies and binding affinities of the Arginase-1 and Arginase-2 reference inhibitors ABH, nor–NOHA and CB-1158 in the literature.

Inhibitor	Human Arginase-1	Human Arginase-2	Method (reference)
ABH	*K*_D_ = 5 nM		ITC at pH 8.5 (Di Costanzo et al., 2005)
	*K*_D_ = 18 nM		SPR at pH 8.5 (Ilies et al., 2011)
	IC_50_ = 1.45–1.55 µM	IC_50_ = 1.92–2.55 µM	Enzyme assay at pH 7.4 (Golebiowski et al., 2013, Van Zandt et al., 2013, Golebiowski et al., 2013, Van Zandt et al., 2019) a
		*K*_i_ = 0.25 µM	Enzyme assay at pH 7.5 (Colleluori and Ash, 2001)
		*K*_i_ = 8.5 nM	Enzyme assay at pH 9.5 (Colleluori and Ash, 2001)
Nor-NOHA	*K*_D_ ≈ 50 nM		ITC at pH 8.5 (Di Costanzo et al., 2010)
	*K*_D_ = 517 nM		SPR at pH 8.5 (Di Costanzo et al., 2010)
		*K*_i_ = 51 nM	Enzyme assay at pH 7.5 (Colleluori and Ash, 2001)
	IC_50_ = 1.36 µM	IC_50_ = 1.26 µM	Enzyme assay at pH 7.4 (Van Zandt et al., 2019)
CB-1158	IC_50_ = 86 nM	IC_50_ = 296 nM	Enzyme assay at pH 7.4 (Oberlies et al., 2009)

a Refs. (Van Zandt et al., 2013, Golebiowski et al., 2013, Van Zandt et al., 2019) are inconclusive about whether the *R*-isomer or racemic ABH is used, despite reporting identical or highly similar IC_50_ values. The use of racemic ABH can be assumed based on comparison to Ref. (Golebiowski et al., 2013).

In this work, we characterized the biochemical potency of the reference inhibitors ABH and nor-NOHA, and the clinical compound CB-1158, side-by-side. Differences in pH-dependent inhibition profiles were further studied in a thermal stability assay and by surface plasmon resonance (SPR). A crystal structure of Arginase-1 in complex with ABH at pH 7.0 and 9.0 shows the determinants of the alkaline pH optimum of Arginase-1. Finally, we demonstrate the high potency of the clinical compound CB-1158 (Fig. 1) (fdasis.nlm.nih.gov/srs/auto/cb-1158) (Geiger et al., 2016), display its slow association and dissociation kinetics and reveal a crystal structure of CB-1158 bound in the Arginase-1 active site.

## 2 Results

### 2.1 Enzyme kinetics and thermal stability of Arginase-1

To enable characterization of the inhibitors in biochemical assays, human Arginase-1 was expressed in *Escherichia coli* with an N-terminal *hexa*-histidine tag and purified by affinity chromatography to > 95% purity (Fig. S1). Enzyme kinetics of the Arginase-1 preparation were characterized by determining the apparent Michaelis-Menten parameters both at physiological pH 7.4 and at the pH optimum of 9.5 using a colorimetric urea assay (Jung et al., 1975, Zawada et al., 2009). At pH 7.4, a Michaelis constant (*K*_M_) of 2.3 mM and a catalytic rate constant (*k*_cat_) of 57 s^−1^ for the substrate L-arginine were measured, while at pH 9.5, *K*_M_ was 4.9 mM and *k*_cat_ was 4.6 × 10^2^ s^−1^. These values correspond well with *K*_M_ values of 1.9, 2.3 and 1.5 mM and *k*_cat_ values of 3.0 × 10^2^ and 1.9 × 10^2^ s^−1^ reported in the literature (Stone et al., 2010, Alarcón et al., 2006, Tsui et al., 2009), despite differences in the pH value and temperature used during determination. The 8-fold increase in *k*_cat_ and 4-fold increase of the specificity constant *k*_cat_/*K*_M_ at pH 9.5 compared to pH 7.4 demonstrate the pH dependence of the Arginase-1 enzyme kinetics and underline the remarkable decrease in catalytic activity at physiological, but non-optimal pH.

**Fig. 2:**
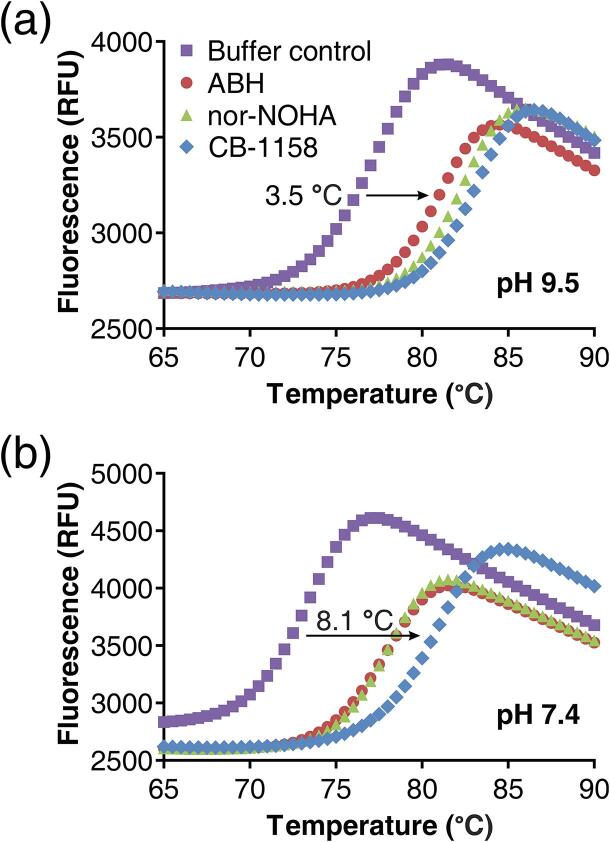
Stabilization of Arginase-1 from thermal unfolding by inhibitors in a thermal shift assay. (a) Representative melting curves of Arginase-1 in the presence of ABH, nor-NOHA and CB-1158 at pH 9.5 and (b) at pH 7.4. The arrow in both graphs indicates an example shift of the midpoint of unfolding.

We reasoned that the higher enzymatic activity of Arginase-1 at pH 9.5 compared to pH 7.4 could be related to an altered stability of the enzyme at pH 9.5. To investigate this further, we developed a thermal shift assay and determined the melting temperature (*T*_m_) of unliganded Arginase-1 at both pH conditions relevant for Arginase-1 activity, *i.e.*, pH 9.5 and 7.4. The melting temperature at pH 7.4 was 72.9 °C, whereas at pH 9.5 it was 77.4 °C (Fig. 2 and Table 2; Buffer control). This difference is significant as is apparent from the low standard deviations (Table 2). Moreover, these values are similar to the *T*_m_ reported for rat Arginase-1 of 75 °C at pH 7.5 (Scolnick et al., 1997), and are slightly lower than the *T*_m_ of 81.0 °C previously reported for recombinant human Arginase-1 at pH 7.4 (Romero et al., 2012), thereby confirming the remarkable thermal stability of Arginase-1 (Scolnick et al., 1997, Romero et al., 2012). Thus, Arginase-1 is not only less active at pH 7.4 compared to pH 9.5 (as determined from the apparent Michaelis-Menten parameters), but also has a significantly decreased thermal stability.

**Table 2:** Inhibition constants in the Arginase-1 colorimetric urea assay and effects on Arginase-1 thermal stability at pH 9.5 and 7.4.

		Inhibition assay		Thermal shift assay		
	Inhibitor	IC_50_ (nM) (95% CI)^a^	*K*_i_ (nM) (95% CI)^a^	*T*_m_ (°C)	SD *T*_m_	Δ*T*_m_ (°C)
pH 9.5	Buffer control			77.4	0.13	–
	ABH	22 (19 – 26)	11 (9.4 – 13)	80.9	0.09	3.5
	nor-NOHA	109 (91 – 130)	54 (45 – 64)	82.1	0.10	4.7
	CB-1158	132 (109 – 159)	65 (54 – 79)	83.0	0.11	5.6

pH 7.4	Buffer control			72.9	0.13	–
	ABH	184 (171 – 198)	88 (82 – 95)	77.4	0.10	4.5
	nor-NOHA	59 (50 – 70)	28 (24 – 34)	77.7	0.04	4.8
	CB-1158	8.6 (8.4 – 8.9)	4.1 (4.0 – 4.3)	81.0	0.23	8.1

a Average IC_50_ and *K*_i_ values and 95% confidence intervals (95% CI) were determined using respectively the pIC_50_ and p*K*_i_ values of the individual experiments.

### 2.2 Inhibition of Arginase-1 by ABH and CB-1158 is pH-dependent

To elucidate the role of pH in Arginase-1 inhibition, we compared inhibitor dose-response curves in the colorimetric urea assay for Arginase-1 activity at pH 7.4 and 9.5 (Table 2 and Fig. S2). ABH, nor-NOHA and CB-1158 are all potent Arginase-1 inhibitors with low- to mid-nanomolar potencies at both pH 9.5 and 7.4. Since the activity assay-based IC_50_ values in literature are generally determined at pH 7.4, and the binding assay-based *K*_D_ values at pH 8.5 (Table 1), we can best compare our values at pH 7.4 with the literature data.

For ABH, the activity assay-based *K*_i_ value of 88 nM lies in between the reported *K*_D_ values of 5 and 18 nM, and IC_50_ values of 1.45–1.55 µM (Table 1, Table 2) (Golebiowski et al., 2013, Van Zandt et al., 2013, Golebiowski et al., 2013, Van Zandt et al., 2019, Di Costanzo et al., 2005, Ilies et al., 2011). For nor-NOHA, the *K*_i_ value is 28 nM at pH 7.4, which is similar to the previously reported *K*_D_ of 50 nM based on isothermal titration calorimetry (Di Costanzo et al., 2010), but deviates considerably from the *K*_D_ of 517 nM reported in the same publication based on SPR experiments (Di Costanzo et al., 2010) and deviates even further from its reported IC_50_ value of 1.36 μM (Table 1, Table 2) (Van Zandt et al., 2019). CB-1158, with an activity assay-based *K*_i_ value of 4.1 nM at pH 7.4, is considerably more potent than previously reported (IC_50_ of 86 nM; Table 1, Table 2) (Steggerda et al., 2017).

Comparison of the *K*_i_ values at the two pH values demonstrates that the potency of nor-NOHA is only slightly affected by an increase of the pH from 7.4 to 9.5 (Table 2). In contrast, an increase of the pH from 7.4 to 9.5 results in an 8-fold decrease in the *K*_i_ of ABH from 88 to 11 nM, whereas the *K*_i_ of CB-1158 contrastingly increases 16-fold from 4.1 to 65 nM (Table 2).

### 2.3 Arginase-1 is thermally stabilized by inhibitors

Next, we studied the effect of the three inhibitors on the Arginase-1 melting temperature at both pH values. We found that all three inhibitors clearly increase the *T*_m_ of Arginase-1 (Fig. 2 and Table 2). Melting temperature shifts (Δ*T*_m_) varied from 3.5 to 8.1 °C, with the largest shift of 8.1 °C observed for CB-1158 at pH 7.4. Thus, the binding of Arginase-1 inhibitors can be measured using thermal shift analysis. At both pH values, the rank order of the inhibitor-induced melting temperature shifts is identical. ABH induces the least stabilization of Arginase-1 followed by nor-NOHA and CB-1158, which increasingly stabilize the enzyme (Table 2). This rank order is identical to the potency rank order in the biochemical activity assay at pH 7.4, while at pH 9.5, the rank order opposes that of the biochemical assay (Table 2). Therefore, inhibitory potency and thermal stabilization are correlated at pH 7.4, but not at pH 9.5.

### 2.4 ABH and CB-1158 display slow association and dissociation kinetics

**Fig. 3:**
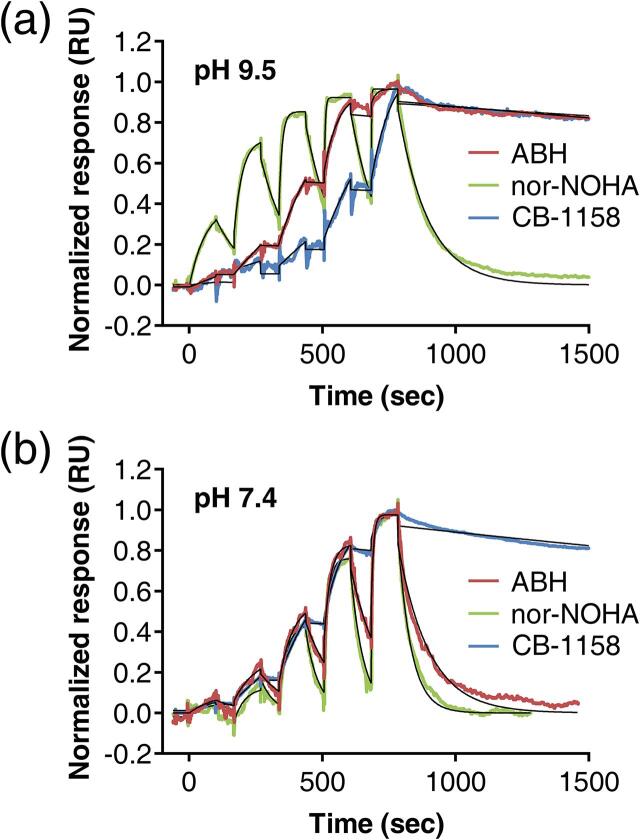
Surface plasmon resonance sensorgrams of Arginase-1 inhibitors. (a) Binding of inhibitors to immobilized Arginase-1 at pH 9.5 and (b) at pH 7.4 measured using single-cycle kinetics. The data used for curve fitting are truncated to the time point when the baseline level is reached. The colored lines show the actual response determined by SPR, while the black lines display the fits obtained using a 1:1 binding model. Individual graphs with absolute responses and a longer time scale (for the inhibitors with long target residence times) can be found in Fig. S3.

To further characterize the inhibitors, we determined the kinetic parameters of their association and dissociation by SPR (Fig. 3 and Table 3). The association rate constants (*k*_a_) of all three inhibitors at pH 9.5 and 7.4 are substantially below the typical diffusion-controlled limit of ~10^8^–10^9^ M^−1^ s^−1^ (Copeland et al., 2006). CB-1158 at pH 9.5 shows the slowest formation of the enzyme-inhibitor complex having a *k*_a_ of 1.3 × 10^3^ M^−1^ s^−1^. The dissociation rate constants (*k*_d_) vary up to 200-fold among the inhibitors and pH conditions (Table 3), with CB-1158 at pH 9.5 also having the slowest dissociation kinetics with a *k*_d_ of 9.2 × 10^−5^ s^−1^ and a target residence time (τ) of 3 h (*i.e.*, 11,000 s). Moreover, two other conditions show particularly slow kinetics. At pH 9.5, ABH has a *k*_a_ of 5.1 × 10^3^ M^−1^ s^−1^, and its dissociation kinetics (*k*_d_ of 1.4 × 10^−4^ s^−1^) are only slightly faster than those of CB-1158 at the same pH. Additionally, CB-1158 at pH 7.4 has *k*_a_ and *k*_d_ values of respectively 4.8 × 10^3^ M^−1^ s^−1^ and 1.8 × 10^−4^ s^−1^ (Table 3). Nor-NOHA has the fastest association and dissociation kinetics at both pH values.

**Table 3:** Kinetic parameters of inhibitor binding to Arginase-1 determined by SPR at pH 9.5 and 7.4.

	Inhibitor	*k*_a_ (M^−1^ s^−1^)	log(*k*_a_)	SD log(*k*_a_)	*k*_d_ (s^−1^)	log(*k*_d_)	SD log(*k*_d_)	*K*_D_ (nM)	τ (s)	No. exp^a^
pH 9.5	ABH	5.1 × 10^3^	3.71	0.03	1.4 × 10^−4^	−3.86	0.03	27	7200	3
	nor-NOHA	3.6 × 10^4^	4.55	0.15	6.2 × 10^−3^	−2.21	0.15	173	160	3
	CB-1158	1.3 × 10^3^	3.11	0.04	9.2 × 10^−5^	−4.04	0.04	72	11,000	3

pH 7.4	ABH	1.2 × 10^4^	4.09	0.21	9.9 × 10^−3^	−2.00	0.21	797	100	6
	nor-NOHA	1.2 × 10^4^	4.09	0.21	1.9 × 10^−2^	−1.73	0.21	1497	54	6
	CB-1158	4.8 × 10^3^	3.68		1.8 × 10^−4^	−3.74		38	5500	2

a Number of experiments

We compared our data to the previously reported SPR-based *K*_D_ values of ABH and nor-NOHA determined at pH 8.5 (Table 1) (Ilies et al., 2011, Di Costanzo et al., 2010). For ABH, the *K*_D_ of 27 nM at pH 9.5 is similar to the reported *K*_D_ of 18 nM (Ilies et al., 2011), while the *K*_D_ of 797 nM at pH 7.4 deviates considerably from this value (Table 1, Table 3). For nor-NOHA, the previously reported *K*_D_ value of 517 nM lies neatly between our *K*_D_ values of 173 nM and 1497 nM at pH 9.5 and 7.4, respectively (Table 1, Table 3) (Di Costanzo et al., 2010).

### 2.5 Structure of the Arginase-1/ABH complex shows an active site-bound sodium ion

To investigate the structural basis of the pH-dependent changes in both Arginase-1 enzyme kinetics and inhibitor potencies, we set up crystallization experiments with the same Arginase-1 enzyme, containing the intact *hexa*-histidine tag and linker, as used in the biochemical assays (Fig. S1a). After initial screening, we discovered crystallization conditions at a low pH of 4.0 resulting in crystals belonging to space group P6_3_, a space group not previously reported for human Arginase-1 according to the Protein Data Bank (PDB). These crystals were formed in the absence of the problematic hemihedral twinning growth defect reported for all human Arginase-1 crystal structures in the literature. However, when we increased the pH of the formed crystals and soaked them with inhibitors, the hemihedral twinning problem returned and the crystals shifted to the apparent space group P3. Upon solving the crystal structures, we noticed that this is presumably due to changes in the crystal contacts between intact Arginase-1 homotrimers.

We soaked the crystals with the inhibitor ABH, since this is the most extensively studied Arginase-1 inhibitor in the literature. Despite the twinning problem, we obtained high-quality data of the crystal structure of the Arginase-1/ABH complex (Table 4). Although a previous structure of this complex at pH 6.5 existed in the PDB with a resolution of 1.29 Å (hereafter referred to by its PDB ID: 2AEB) (Di Costanzo et al., 2005), we wanted to look specifically at the effect of the pH on the Arginase-1 crystal structure and ABH binding. Therefore, we prepared and measured two crystals under identical conditions, with the only exception being the pH used during soaking with ABH. Both crystal structures have a high resolution of respectively 1.50 and 1.66 Å at pH 7.0 and 9.0 (Table 4).

**Table 4:** Data collection and refinement statistics.

PDB ID	6Q92	6Q9P	6QAF
Ligand	ABH	ABH	CB-1158
pH	7.0	9.0	9.0
*Data collection*^a^			
Beamline	ID30A-1 (ESRF)	ID30A-1 (ESRF)	ID30A-1 (ESRF)
Wavelength (Å)	0.966	0.966	0.966
Space group	P 3	P 3	P 3
Unit cell parameters			
*a, b, c* (Å)	90.4, 90.4, 69.3	90.0, 90.0, 69.1	90.1, 90.1, 69.1
*α, β, γ* (°)	90, 90, 120	90, 90, 120	90, 90, 120
Resolution limits (Å)^b^	39.14 – 1.50 (1.53 – 1.50)	44.99 – 1.66 (1.69 – 1.66)	51.74 – 1.61 (1.64 – 1.61)
Total reflections	238,075 (9936)	174,685 (8764)	160,083 (8063)
Unique reflections	99,464 (4790)	73,201 (3621)	79,090 (3888)
Mean *I/σ(I)*	6.9 (1.8)	6.0 (1.2)	6.9 (1.1)
Completeness (%)	98.2 (96.4)	98.9 (97.5)	96.9 (96.0)
*R*_merge_	0.097 (0.608)	0.098 (0.736)	0.058 (0.658)
*R*_pim_	0.074 (0.508)	0.077 (0.587)	0.049 (0.561)
CC_1/2_^c^	0.987 (0.293)	0.971 (0.385)	0.996 (0.368)
Wilson B-factor (Å^2^)	12.2	21.6	20.7

*Refinement*^d^			
*R*_work_/*R*_free_ (%)	13.5/14.7	14.8/17.8	14.7/17.7
No. of test reflections	4377 (4.3%)	3816 (5.2%)	4210 (5.2%)
No. of atoms	5486	5184	5254
Average B-factor (Å^2^)	17	25	25
Ramachandran plot (%)			
Favored	96.7	96.9	96.8
Allowed	3.0	2.6	2.8
Outliers	0.3	0.5	0.3
RMSD bond lengths (Å)	0.007	0.011	0.009
RMSD bond angles (°)	1.306	1.524	1.357

a Data reduction statistics were calculated using the program Aimless (version 0.7.1) in the CCP4i2 package (Winn et al., 2011).

b Values in parentheses are for the highest resolution shell. Data in this resolution shell were used to calculate the statistics in parentheses in the lines below.

c Correlation coefficient between equivalent reflections. A value of > 0.3 in the last resolution shell was used to determine the resolution cut-off.

d Data refinement statistics are reported as determined by the Protein Data Bank validation pipeline, or the program Refmac5 (version 5.8.0218) in the CCP4i2 package (Winn et al., 2011).

**Fig. 4:**
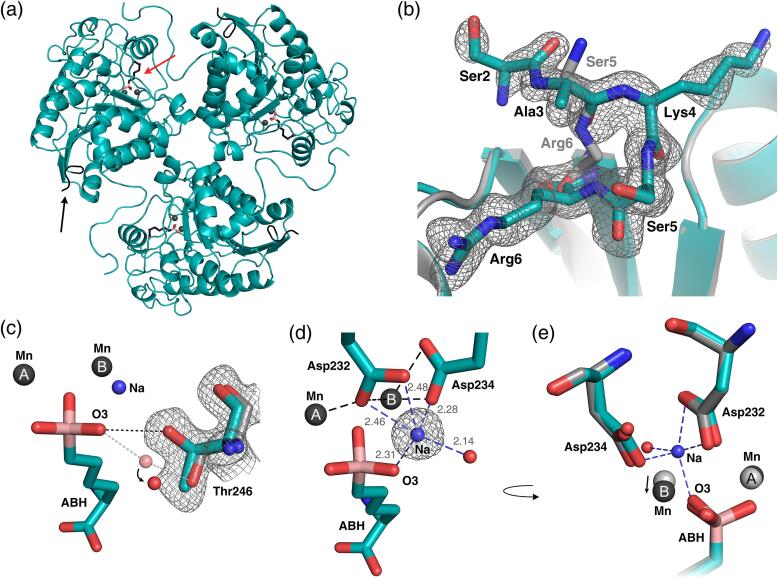
Overlay of the crystal structure of the Arginase-1/ABH complex at pH 7.0 (PDB ID: 6Q92; cyan) and the previously reported Arginase-1/ABH crystal structure (PDB ID: 2AEB; grey) (Di Costanzo et al., 2005). (a) Overview of structure 6Q92 showing the trimeric quaternary structure of Arginase-1. The red arrow indicates the location of the active site into which ABH is bound. The black arrow indicates the N-terminal residues Ser2 to Arg6. (b) The N-terminal residues Ser2 to Arg6 of the human Arginase-1/ABH complex at pH 7.0. Electron density around the N-terminal residues is displayed as an 2*F*_obs_-*F*_calc_ omit map contoured at 1.0 σ (standard deviation of electron density). The previously reported human Arginase-1/ABH crystal structure 2AEB is displayed in grey for only the backbone atoms. (c) Active site structure focusing on the alternate conformation of Thr246 compared to the structure 2AEB (grey with water molecule displayed in light red). Electron density is displayed as in panel b. (d) Observed electron density for a sodium ion in the active site and its coordination interactions. Electron density is displayed as in panel b. (e) Different orientation of the Asp232 and Asp234 residues, and the different position of the manganese ions between our structure (dark grey) and 2AEB (light grey).

In our crystal structure of the Arginase-1/ABH complex at pH 7.0 (PDB ID: 6Q92), Arginase-1 exists as a homotrimer with ABH bound as a tetrahedral boronate anion to the manganese cluster in the active site (Fig. 4a). This form of ABH, which mimics the tetrahedral intermediate in the hydrolysis mechanism of Arginase-1 (Cox et al., 1999), was also observed in previous structures of Arginase-1 in complex with ABH, for example 2AEB (Di Costanzo et al., 2005).

During initial refinement of the Arginase-1/ABH crystal structure at pH 7.0, we noticed the presence of substantial difference density in the *F*_obs_-*F*_calc_ map around the N-terminal residues Ser5 and Arg6. This indicated that these residues were present in a different conformation compared to the structure 2AEB. In addition, the electron density revealed the conformation of the N-terminal residues Ser2, Ala3 and Lys4 (Fig. 4b). We expect that the presence of the purification tag has reduced the flexibility of the N-terminus and allowed visualization of these N-terminal residues of Arginase-1 for the first time. Comparison of the active site of our crystal structure and 2AEB further reveals that in our complex the hydroxyl group of residue Thr246 is pointed towards the O3 hydroxyl group of ABH and can interact directly with this group through a hydrogen bond (Fig. 4c). In the structure 2AEB, the Thr246 hydroxyl group is pointed away from the O3-hydroxyl of ABH and binds it only via a water-mediated hydrogen bond. Since Thr246 forms a direct hydrogen bond to ABH in our structure, this is accompanied by a displacement of the water molecule (Fig. 4c).

During refinement of the Arginase-1/ABH crystal structure at pH 7.0, we also observed a strong difference density in the *F*_obs_-*F*_calc_ map, consistent with the presence of water, or an ion with a low molecular mass, close to the manganese cluster (Fig. 4d). The distances between the center of this electron density and most surrounding atoms were too short for hydrogen-bond interactions but consistent with metal-ion solvation (Fig. 4d). The negative charge of the surrounding Asp232 and Asp234 residues as well as the boronate anion of ABH (Fig. 4d) indicated that the ion carried a positive charge. Since the distances to most surrounding atoms were close to the ideal Na-O distance of 2.41 Å (Fig. 4d) (Zheng et al., 2017), we suspected the presence of a sodium ion. The ion valence determined using the CheckMyMetal web server (Zheng et al., 2017) is consistent with the monovalency of sodium, as is the five-coordinate geometry (Zheng et al., 2017) observed for the ion (Fig. 4d). Sodium was also the only metal present in a significant concentration in the crystallization solution. While the monovalent sodium ion is not generally surrounded by more than one negatively charged carboxyl side chain (Zheng et al., 2017), as we observed for the ion, this can be explained by the additional coordination of these side chains (*i.e.,* Asp232 and Asp234) to the manganese ions (Fig. 4d). Thus, we conclude that the binding of ABH to Arginase-1 introduces the binding of an additional sodium ion in the active site.

A consequence of the presence of a sodium ion close to the manganese cluster is that the Mn_B_^2+^-ion is displaced with respect to the Mn_A_^2+^-ion and the protein, when compared to structure 2AEB (Fig. 4e). This is accompanied by an increase in the Mn_A_^2+^-Mn_B_^2+^ distance from 3.33 Å to 3.43 Å. Moreover, the Asp232 and Asp234 side chains have shifted considerably with respect to the manganese cluster (Fig. 4e). These observations might be explained by both the manganese ions and the sodium ion aiming to achieve ideal coordination geometry. Since the manganese cluster and the sodium ion share the Asp232 and Asp234 residues as coordinating ligands, a displacement of the Mn_B_^2+^-ion as well as a different orientation of the Asp232 and Asp234 residues are needed to achieve the most optimal coordination geometry for all metal ions.

Notably, we only observe the presence of the sodium ion in Arginase-1 crystal structures with boron-containing ligands, including our complexes with ABH at pH 7.0 and 9.0 and our complex with CB-1158 (see below), but not with other types of ligands or the unliganded enzyme. This suggests that these boron-containing ligands bind in a sodium-dependent manner. Interestingly, the sodium ion is not observed in previous Arginase-1/ABH structures, either from human or rat Arginase-1 (PDB ID: 2AEB and 1D3V), although this might be explained by the fact that there was no sodium included in the crystallization conditions used to prepare these crystals (Di Costanzo et al., 2005, Cox et al., 1999). Unfortunately, the original diffraction data were not deposited for these structures, precluding a check if this sodium ion was missed from the electron density in these structures.

### 2.6 pH-dependent structural changes in the Arginase-1/ABH complex

**Fig. 5:**
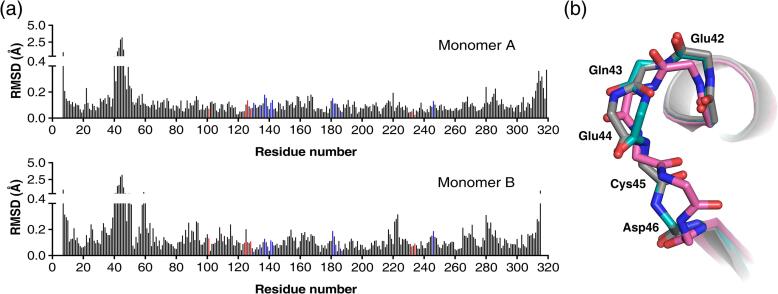
Comparison of the overall structure of the Arginase-1/ABH complexes at pH 7.0 and 9.0. (a) Root-mean-square deviation (RMSD) between the main chain atoms (Cα, C, O and N) of the pH 7.0 complex (PDB ID: 6Q92) and the pH 9.0 complex (PDB ID: 6Q9P) calculated per residue. Red bars indicate manganese-coordinating residues, while blue bars indicate active site residues interacting with ABH. (b) Superposition of the backbone atoms of the Glu42 to Asp46 surface loop in the two structures (pH 7.0 in cyan and pH 9.0 in magenta) and the previously reported Arginase-1/ABH crystal structure (PDB ID: 2AEB; grey).

Comparison of the Arginase-1/ABH complexes at pH 7.0 and 9.0 (PDB IDs: 6Q92 and 6Q9P) yielded a root-mean-square deviation (RMSD) of all main chain atoms of 0.31 Å for superposition of the 313 residues of monomer A and 0.33 Å for the 309 residues of monomer B. This degree of deviation is primarily caused by a change in the surface loop consisting of residues Glu42 to Asp46 (Fig. 5a), which can adopt different conformations (Fig. 5b). This loop appears to be relatively flexible with high average B-factors of 28 Å^2^ at pH 7.0 and 48 Å^2^ at pH 9.0, compared to the lower overall B-factors of the protein of 17 Å^2^ and 25 Å^2^, respectively (Table 4). Omission of these residues from the RMSD calculation results in values of 0.13 Å and 0.17 Å for, respectively, superposition of monomers A and B, indicating that there are no other major conformational changes occurring upon increase of the pH.

**Fig. 6:**
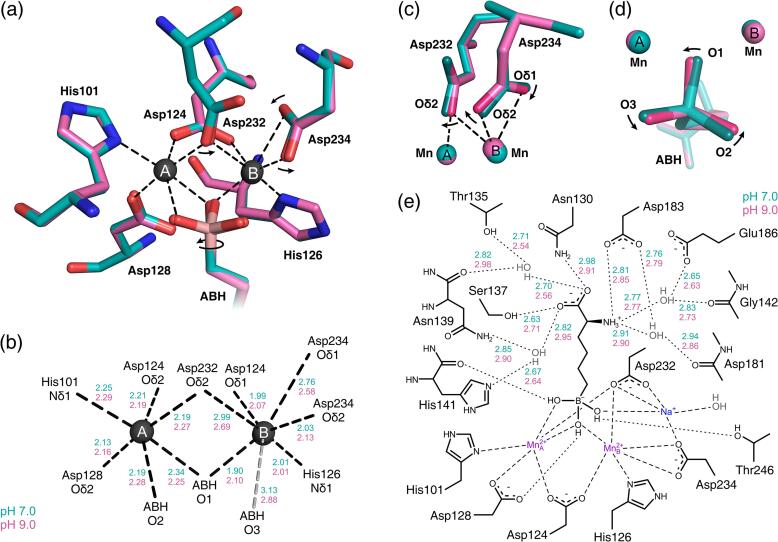
Structural differences between the active sites of the Arginase-1/ABH complexes at pH 7.0 (cyan) and 9.0 (magenta). (a) Active site detail of the superposition of structures at pH 7.0 (PDB ID: 6Q92) and pH 9.0 (PDB ID: 6Q9P). The black dashed lines indicate the coordination interactions of the complex at pH 9.0. The arrows indicate the direction of the most prominent changes in the manganese coordination structure going from pH 7.0 to 9.0. (b) Manganese coordination distances corresponding to the interactions displayed in panel a. Ideal Mn-O distances are 1.91 and 2.19 Å, while ideal Mn-N distances are 1.99 and 2.29 Å (Zheng et al., 2017). The grey dashed line displays an interaction that is not an inner-sphere coordination interaction and is not shown in panel a. (c) Detail of the Asp232 and Asp234 residues coordinating to the manganese cluster. The arrows indicate the direction of the structural changes going from pH 7.0 to 9.0. (d) Detail of the ABH boronate anion bound near the manganese cluster, displayed as in panel c. (e) Schematic representation of ABH bound in the Arginase-1 active site. Dashed lines indicate coordination interactions with the metal ions, while dotted lines indicate hydrogen-bond interactions made by ABH.

Comparison of the active site residues in the manganese coordination structure shows that residues Asp232 and Asp234 undergo the most notable pH-dependent changes (Fig. 6a-c). At pH 7.0, the Asp232-Oδ2 atom is strongly coordinated to Mn_A_^2+^, but has a distance to Mn_B_^2+^ that is too long to be considered inner-sphere metal coordination (Fig. 6b). Upon increase of the pH from 7.0 to 9.0, the Asp232-Oδ2 atom shifts away from Mn_A_^2+^ and towards Mn_B_^2+^, and thereby forms an inner-sphere metal coordination interaction with Mn_B_^2+^ and bridges the manganese ions more symmetrically (Fig. 6a-c). The side chain of the nearby Asp234 also moves upon increase of the pH, resulting in a stronger coordination of the Asp234-Oδ1 atom to Mn_B_^2+^, while coordination of the Asp234-Oδ2 atom to Mn_B_^2+^ is slightly weakened (Fig. 6a-c). These results indicate that the manganese coordination structure of Arginase-1 is more symmetrical and forms stronger bonds at pH 9.0 compared to pH 7.0.

Although ABH binds in similar fashion in both structures, a clear change in its binding mode is observed upon increase of the pH from 7.0 to 9.0. The boronate anion of ABH is rotated with respect to the manganese ions upon increase of the pH. As a result, the coordination distance of the ABH-O2 atom to Mn_A_^2+^ has lengthened, while the distance between the ABH-O3 atom and Mn_A_^2+^ has become shorter at pH 9.0 compared to pH 7.0 (Fig. 6a, 6b and 6d). Moreover, the ABH-O1 atom adopts a more symmetrical coordination to the manganese cluster at pH 9.0 by shortening of its distance to Mn_A_^2+^ and lengthening its distance to Mn_B_^2+^ (Fig. 6b). In the remaining part of the ABH structure, the hydrogen bond interactions of the α-amino group remain practically unchanged, while the hydrogen-bond interactions of the α-carboxylate group are more sensitive to a change of the pH (Fig. 6e). Thus, the pH-dependent difference in the binding mode of ABH is focused mostly around the boronate anion adopting a more symmetrical and therefore more ideal coordination structure to the manganese cluster (Fig. 6a, 6b and 6d).

### 2.7 Binding mode of CB-1158 in the Arginase-1 active site

**Fig. 7:**
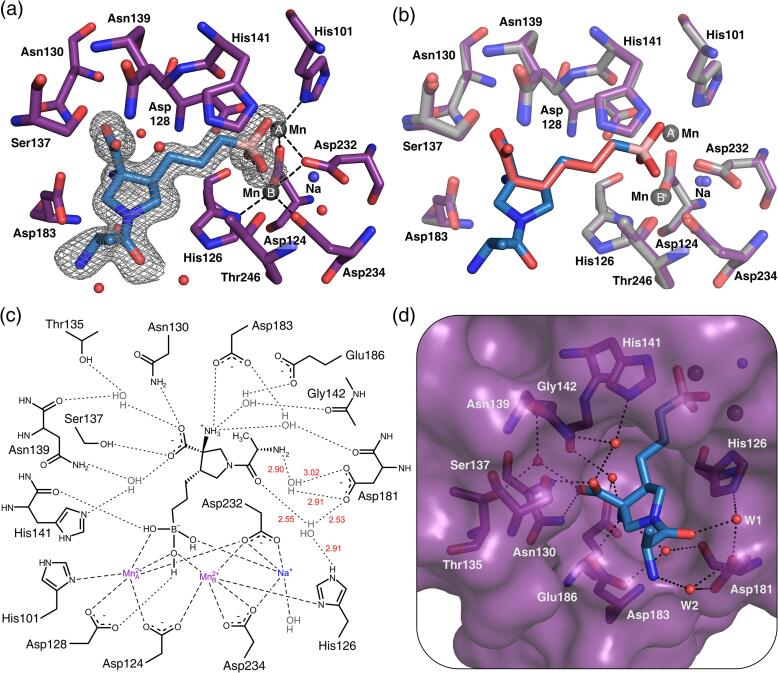
Binding mode of CB-1158 in the human Arginase-1 active site (PDB ID: 6QAF). (a) The Arginase-1 active site containing the inhibitor CB-1158. The electron density surrounding the inhibitor in monomer A is displayed as a 2*F*_obs_-*F*_calc_ omit map contoured at 1.0 σ. The dashed lines indicate coordination interactions of the inhibitor and active site residues with the manganese cluster. Water molecules are displayed as red spheres. (b) Superposition of the crystal structures of the Arginase-1/CB-1158 complex (6QAF, purple and blue) and the Arginase-1/ABH complex at pH 9.0 (PDB ID: 6Q9P; grey and red). (c) Schematic representation of CB-1158 bound in the Arginase-1 active site of monomer A. Dashed lines indicate coordination interactions with the metal ions, while dotted lines indicate hydrogen-bond interactions made by CB-1158. The α-amino group of CB-1158 is displayed in its protonated form, although this group may only be partially protonated at pH 9.0 based on its estimated p*K*_a_ of 9.0. Hydrogen-bond distances of the 2-aminopropanoyl functionality are displayed in red. Thr246 and its hydrogen-bond interaction with CB-1158 are excluded from this figure for reasons of clarity. (d) Orientation of CB-1158 in the active site pocket in monomer A. Water molecules (W) are shown as red spheres. Important hydrogen-bond networks are displayed as dotted lines.

To study the structural basis of the high potency, slow association kinetics, and long target residence time of CB-1158, we determined the crystal structure of human Arginase-1 with CB-1158 at pH 9.0 at a resolution of 1.61 Å (Table 4; PDB ID: 6QAF). The crystal structure shows that the inhibitor binds in the Arginase-1 active site as a tetrahedral boronate anion coordinated to the manganese cluster (Fig. 7a). Superposition with our crystal structure of the Arginase-1/ABH complex at pH 9.0 (PDB ID: 6Q9P) shows that the two inhibitors align nearly perfectly in the active site (RMSD of 0.17 Å for the 13 matching atoms; Fig. 7b). Binding of CB-1158 does not cause any significant conformational changes in the Arginase-1 active site when compared to the binding of ABH (Fig. 7b), and the structure also shows the presence of the active site-bound sodium ion. Moreover, the three direct and four water-mediated hydrogen bonds made by the α-carboxylate and α-amino substituents of ABH (Fig. 6e) are likewise maintained for CB-1158 (Fig. 7c).

The additional 2-aminopropanoyl-functionalised pyrrolidine ring of CB-1158 protrudes from the active site pocket towards the bulk solvent (Fig. 7d), which is in contrast with the tight embedding of the entire structure of ABH in the Arginase-1 active site. The methyl group of the 2-aminopropanoyl functionality of CB-1158 is pointed towards the same side of the pyrrolidine ring as the α-carboxylate on the opposite side of the ring (Fig. 7a and d). In monomer A of our structure, we observe two important water molecules (W1 and W2 in Fig. 7d) in close proximity to this 2-aminopropanoyl functionality. In monomer B of the asymmetric unit, we did not observe clear electron density for the outermost water molecule (W2), which is why we focus on monomer A to describe the full binding potential of CB-1158. In this monomer, the presence of the two water molecules allows for two indirect hydrogen-bond interactions to be made with the enzyme, which are not seen in the ABH complex. The water molecule W1 is involved in an indirect hydrogen bond between the oxygen atom of the amide functionality, and both the surface-exposed Asp181 residue and the active site-lining His126 residue (Fig. 7c and 7d), while W2 interacts solely with the side chain of Asp181. The opportunity to form new hydrogen-bond interactions is likely to contribute to the favorable potency and binding kinetics of CB-1158 compared to ABH (Table 2, Table 3).

## 3 Discussion

The role of Arginase-1 in tumor immune suppression and its potential as a drug target for cancer immunotherapy has culminated in the clinical development of CB-1158 (Steggerda et al., 2017). Given the importance of Arginase-1 inhibitors, we studied the characteristics of ABH, nor-NOHA and CB-1158 side-by-side in different biochemical and biophysical assays, including SPR and protein crystallography.

The biochemical activity assay shows that the most potent inhibition of Arginase-1 occurs by CB-1158 at pH 7.4 (IC_50_ = 4.1 nM; Table 2), which is respectively 21- and 6.8-fold more potent than ABH and nor-NOHA at this pH (Table 2). Moreover, nor-NOHA has a potency which is intermediate to the potencies of ABH and CB-1158 at both pH values, which indicates that the boronic acid warhead is not a requisite for strong Arginase-1 inhibition (Fig. 1). Comparison of the inhibitor potencies at pH 7.4 and 9.5 demonstrates that the pH is an important determinant for the potency of ABH and CB-1158, while nor-NOHA remains mostly unaffected by the pH. Interestingly, ABH and CB-1158 have opposing pH-dependent inhibition profiles, with ABH being less potent at pH 7.4 compared to pH 9.5, while CB-1158 becomes more potent (Table 2). The 8-fold decrease in potency of ABH is in line with a previous study showing that the potency of ABH for human Arginase-2 is almost 30 times lower at pH 7.5 compared to pH 9.5 (Table 1) (Colleluori and Ash, 2001).

In the thermal shift assay, CB-1158 is the strongest stabilizer of Arginase-1 at both pH values (Δ*T*_m_ = 5.6 and 8.1 °C at respectively pH 7.4 and 9.5; Table 2). At pH 7.4, the melting temperature shift induced by CB-1158 (Δ*T*_m_ = 8.1 °C) is even more than 3 °C stronger than the shifts induced by ABH and nor-NOHA (Δ*T*_m_ = 4.5 and 4.8 °C, respectively; Table 2). For both nor-NOHA and CB-1158, there is a correlation between the activity assay-based inhibitory potencies and the shifts in thermal stability. When comparing both pH values, nor-NOHA has roughly equal potency in the activity assay as well as an equal effect in the thermal shift assay. CB-1158 has a considerably higher potency in the activity assay at pH 7.4 compared to pH 9.5, consistent with its larger shift of thermal stability at pH 7.4 (Table 2).

The inhibitor binding kinetics determined by SPR show that CB-1158 has both the slowest association and the slowest dissociation kinetics among the inhibitors (Table 3). We expect that these slow dissociation kinetics contribute to its favorable inhibitory potency in the activity assay, which is especially observed at pH 7.4 (Table 2), since the equilibrium binding constant is inversely related to the dissociation constant. For ABH, we observe slow association and dissociation kinetics only at pH 9.5, while its kinetics at pH 7.4 are similar to the relatively fast kinetics of nor-NOHA (Table 3). The slower dissociation of ABH at higher pH is consistent with its increased inhibitory potency at pH 9.5 compared to pH 7.4.

The side-by-side comparison of ABH, nor-NOHA and CB-1158 in different biochemical assays shows that CB-1158 has overall the most favorable characteristics at both pH values, thereby supporting its potential as a drug for targeting tumor immune suppression. Nonetheless, it should be noted that nor-NOHA performed most consistently among the two pH values in the different assays. Therefore, despite its poor pharmacokinetic properties (Havlinova et al., 2013), nor-NOHA may still prove valuable as a tool compound for studying Arginase-1 *in vitro*. Moreover, the relatively potent activity of nor-NOHA indicates that potent Arginase-1 inhibitors without a boronic acid warhead can be developed.

In order to understand the pH-dependent effects of Arginase-1 inhibition and stabilization, we studied the pH optimum of Arginase-1. We measured a roughly 8-fold higher catalytic rate constant (*k*_cat_) of Arginase-1 at pH 9.5 compared to pH 7.4, which is consistent with the frequently reported alkaline pH optimum of the enzyme (Zakalskiy et al., 2012, Jenkinson et al., 1996, Cabello et al., 1961, Berüter et al., 1978). This pH optimum could simply result from the catalytic mechanism of Arginase-1 requiring a hydroxide ion to be bound in the active site, which is present in higher concentrations at alkaline pH (Scolnick et al., 1997). However, given the general notion that enzymes evolve to function at their physiologically relevant pH, this would suggest that alkaline pH is relevant for the biological function of Arginase-1. For granulocytic Arginase-1, it has been proposed that its enzymatic activity is initiated by a rise in the phagosomal pH up to 8.5–9.5 upon initiation of neutrophil phagocytosis (Munder et al., 2005, Levine et al., 2015). Nevertheless, in the context of cancer, it is known that the extracellular tumor microenvironment is slightly acidic (pH 6.5–7.1), while the intracellular pH of tumors ranges between 7.2 and 7.5 (Schwartz et al., 2017, Webb et al., 2011). In tumors, Arginase-1 may therefore function at alkaline pH during the early phase of phagocytosis (Munder et al., 2005), while it may continue to function at more neutral pH values in the later stages of phagosome maturation and in the extracellular tumor microenvironment. Therefore, alkaline pH values close to the Arginase-1 optimum as well as more neutral pH values may both be relevant for Arginase-1 functioning inside a tumor.

To study the structural basis of the alkaline pH optimum, we determined the crystal structures of the Arginase-1/ABH complex at pH 7.0 and 9.0. We observe a number of small but significant shifts in the manganese coordination structure, which becomes more symmetrical at increased pH (Fig. 6a–d). This probably allows for a more optimal positioning of the hydroxide ion required for Arginase-1 catalytic activity, which is represented in our structures by the O1-atom of ABH (Fig. 6d). The more symmetrical coordination of the manganese cluster presumably underlies the increased activity of Arginase-1 at higher pH (Fig. 6a–d).

In order to understand the increased potency of ABH at increased pH, we looked specifically into the binding mode of ABH in the Arginase-1 active site at pH 7.0 and 9.0. (Fig. 6a, b and d). Since ABH mimics the tetrahedral intermediate in the Arginase-1 catalytic mechanism, we postulate that it may bind better to a catalytically more competent enzyme, *i.e.,* Arginase-1 at pH 9.0. This is substantiated by our crystal structures showing that the boronate anion of ABH assumes a more symmetrical coordination to the manganese cluster at increased pH (Fig. 6b and 6d). Since there are no considerable changes observed in the orientation or the interactions made by the remainder of the ABH structure (Fig. 6e), we expect that this more symmetrical orientation with respect to the manganese cluster is the reason why ABH binds more potently at increased pH.

Interesting is also the observation of the sodium ion in the Arginase-1 active site of our structures, which has not been previously reported for any Arginase-1 crystal structure. Since this sodium ion is located in such close proximity to the manganese cluster (Fig. 4d), and has a direct influence on the position of the Mn_B_^2+^-ion as well as the orientation of the Asp232 and Asp234 side chains (Fig. 4e), we expect that this sodium ion could prove important in the mechanism of Arginase-1 inhibition by boron-containing inhibitors.

The structural basis of the favorable potency of CB-1158 was uncovered by the crystal structure of Arginase-1 in complex with this inhibitor (Fig. 7). This structure indicates that the binding mode of CB-1158 is consistent with the binding mode of ABH that we observe in our structures. Moreover, an additional hydrogen-bond network involving two water molecules and the residues Asp181 and His126 (Fig. 7) is expected to contribute to its potent inhibition character (Table 2). Water-mediated hydrogen-bond interactions with Asp181 have previously been reported for α,α-disubstituted ABH analogues, and this correlated with a more favorable potency of these analogues compared to ABH (Van Zandt et al., 2013). The crystal structure of CB-1158 is the first to also show a water-mediated hydrogen-bond interaction with the more buried His126 residue. This crystal structure therefore indicates that binding towards these active site residues is beneficial for inhibitor affinity. Moreover, CB-1158 is expected to have an increased rigidity compared to ABH due to a decrease in the number of rotatable bonds by introduction of the pyrrolidine ring (Fig. 1), which constricts CB-1158 into the correct rotamer for binding into the Arginase-1 active site (Fig. 7). We expect that this rigidity also contributes to the potent inhibition of Arginase-1 by CB-1158, as measured in the biochemical activity assays (Table 2).

CB-1158 is more potent at pH 7.4 compared to pH 9.5. The amino group of the 2-aminopropanoyl functionality of CB-1158 (Fig. 1) has a predicted p*K*_a_ value of 8.2, indicating that this group is predominantly protonated at pH 7.4 and unprotonated at pH 9.5. While the amino group is pointed away from the acidic Asp181 residue and forms only a water-mediated hydrogen bond with this residue at pH 9.0 (Fig. 7c and 7d), binding of the inhibitor at pH 7.4 may be favored by electrostatic interaction of the protonated amino group with the active site Asp181 residue. Alternatively, another nearby residue such as the acidic Asp183 (Fig. 7d) may facilitate the positioning of the inhibitor in the Arginase-1 active site at pH 7.4. This may explain the observed increase in potency at lower pH. The increase in potency of CB-1158 contrasts with the decrease in potency observed for ABH at pH 7.4 compared to pH 9.5 (Table 2). The crystal structures show that ABH makes less interactions with the Arginase-1 active site than CB-1158, apart from the boronate anion (Fig. 6e and 7c). This could indicate that the coordination symmetry of the boronate anion to the manganese cluster plays the most significant role in inhibitor binding at pH 9.5, whereas at pH 7.4, the interactions made by the remaining structure of the inhibitor are more important for inhibitor potency.

CB-1158 appears to be an Arginase-1 inhibitor with a long target residence time at both pH 9.5 and 7.4 (Fig. 3 and Table 3), which could be very interesting due to anticipated favorable pharmacokinetic properties (Copeland et al., 2006). Moreover, formation of the enzyme-inhibitor complex appears to occur with slow association. For ABH at pH 9.5, slow association and dissociation kinetics similar to those of CB-1158 are found. While slow-binding inhibitors of Arginase-1 have not previously been reported, this is in agreement with a previous study on Arginase-2 claiming that ABH has a slow-binding character at pH 9.5 (Colleluori and Ash, 2001). This is explained by the fact that at pH 9.5 the tetrahedral boronate form of boron-containing inhibitors is expected to predominate over the trigonal boronic acid form. Slow association of the inhibitor may therefore be caused by a slow conformational change of the active site required to accommodate the tetrahedral boronate species. Additionally, binding of the tetrahedral inhibitor species requires the expulsion of the tightly-bound manganese-coordinated hydroxide ion from the active site, which is replaced by a hydroxyl group of the boronate anion. This displacement is suggested to be a slow event, which could contribute to the slow association kinetics of the inhibitor (Colleluori and Ash, 2001). However, the fact that CB-1158 has slow association and dissociation kinetics at both pH 7.4 and 9.5, while ABH only has comparable slow kinetics at pH 9.5, indicates that another factor contributes to the kinetics of CB-1158. We propose that the slow binding kinetics of CB-1158 are due to, or enhanced by, an active site conformational change. As we do not observe such a change in the crystal structure of the Arginase-1/CB-1158 complex (Fig. 7b), we expect that a conformational change is only a temporary adaptation required for the binding event of the inhibitor, and that the conformation of the enzyme returns to normal once the inhibitor is bound. Such conformational plasticity may only be required for binding of CB-1158 in the active site, but not for ABH or nor-NOHA, because CB-1158 has a larger size and reduced flexibility compared to these inhibitors. This binding mechanism may also explain the long target residence time of CB-1158, since dissociation of the tetrahedral boronate form of the inhibitor from the enzyme active site will require a similar conformational change to take place.

In summary, we show that the alkaline pH optimum of Arginase-1 is not merely a consequence of the higher abundance of hydroxide ions at increased pH, but that Arginase-1 also shows changes at a structural level by the catalytic manganese ions adopting a more symmetrical coordination structure at elevated pH. We have uncovered the contrasting pH-dependence of the potencies of ABH and CB-1158. We propose that at increased pH, the coordination symmetry of the boronate anion to the manganese cluster becomes increasingly important for inhibitor potency compared to interactions made by the remainder of the inhibitor. Using SPR, we show that association and dissociation of CB-1158 from the Arginase-1 active site occurs through slow kinetics. We propose that active site conformational plasticity is involved in inhibitor binding, probably due to its increased rigidity. Finally, comparison of the crystal structures of Arginase-1 bound to ABH and CB-1158 reveals an additional hydrogen-bond network formed by CB-1158, which, in addition to the increased rigidity of this inhibitor, might underly its favorable potency. The crystal structure of the Arginase-1/CB-1158 complex will support future structure-based drug design efforts of Arginase-1 inhibitors.

## 4 Materials and methods

### 4.1 Protein expression and purification of Arginase-1

Full-length human Arginase-1 containing an N-terminal *hexa*-histidine tag and thrombin-cleavable linker (Fig. S1a) was expressed in *Escherichia coli* Rosetta (DE3) competent cells (cat. no. 70954–4; Novagen, Temecula, CA). Bacteria were cultured in LB medium containing 35 μg/mL chloramphenicol and 100 μg/mL ampicillin in a shaking incubator at 37 °C to OD_600_ of 0.6 – 0.8. Expression was induced by addition of 0.1 mM isopropyl-β-D-thiogalactopyranoside, followed by incubation at 37 °C for 3 – 4 h. The cells were harvested by centrifugation and the resulting pellets were stored at −20 °C. For purification, the bacterial pellets were resuspended in purification buffer, consisting of 10 mM HEPES, pH 7.5, 100 mM NaCl, 1 mM TCEP, 1 mM MnCl_2_ and cOmplete™ EDTA-free protease inhibitor cocktail (Roche, Basel, Switzerland). The cells were lysed using a liquid homogenizer (Avestin, Ottawa, ON, Canada). After centrifugation, the supernatant was heated to 60 °C for 20 min and purification was performed by affinity chromatography using Ni-NTA Superflow beads (Qiagen, Venlo, The Netherlands). For use in biochemical assays, thermal shift assays and surface plasmon resonance, the purified protein was desalted on PD-10 columns (GE Healthcare, Buckinghamshire, UK) and supplemented with 20% glycerol (cat no. 158920025; Acros Organics, Geel, Belgium) prior to storage at −80 °C. Purity of the enzyme was estimated by visual inspection of SDS-PAGE gels (Fig. S1b and S1c). For use in protein crystallography, the purification buffer was exchanged to crystallization buffer (50 mM Bicine, pH 8.5, and 100 µM MnCl_2_), followed by concentration of the enzyme to 3.6 mg/mL, as determined using a NanoDrop 2000 Spectrophotometer (Thermo Scientific, Waltham, MA), and storage at −80 °C.

### 4.2 Inhibitors

ABH was purchased from Sigma-Aldrich (cat. no. SML1466; St. Louis, MO), nor-NOHA from Tocris Bioscience (cat. no. 6370; Bristol, UK) and CB-1158 ((3*R*,4*S*)-3-amino-1-[(2*S*)-2-aminopropanoyl]-4-(3-boronopropyl)pyrrolidine-3-carboxylic acid) from ChemieTek (cat. no. CT-CB1158; Indianapolis, IN).

### 4.3 Colorimetric urea assay

Arginase-1 activity was monitored by measurement of the rate of urea formation in a classic colorimetric assay (Jung et al., 1975, Zawada et al., 2009). All components of the assay were diluted in Arginase reaction buffer 1 (8 mM Na_2_HPO_4_, 2 mM KH_2_PO_4_, pH 7.4, 137 mM NaCl, 2.7 mM KCl, and 0.05% Tween-20) or Arginase reaction buffer 2 (10 mM glycine, pH 9.5, 137 mM NaCl, 2.7 mM KCl, and 0.05% Tween-20). Compounds were either dissolved and diluted in DMSO, followed by further dilution in reaction buffer, or dissolved in MilliQ water (MQ) and diluted in reaction buffer to the desired concentrations. In a clear 384-well plate (cat. no. 781101; Greiner), 10 µL diluted compound and 10 µL of 3 nM (pH 9.5) or 15 nM (pH 7.4) Arginase-1 were combined and incubated for 90 min at room temperature. Then, 10 µL of 15 mM (pH 9.5) or 7.5 mM (pH 7.4) L-arginine (cat. no. 105000250; Acros Organics) was added to the plate, followed by incubation for 30 min. A 1:1 mixture of reagent A (10 mM *o*-phthaldialdehyde, 0.4% polyoxyethylene (23) lauryl ether (w/v) and 1.8 M sulfuric acid) and reagent B (1.3 mM primaquine diphosphate, 0.4% polyoxyethylene (23) lauryl ether (w/v), 130 mM boric acid and 3.6 M sulfuric acid) was prepared. The enzymatic reaction was stopped by addition of 30 μL of the reagent A + B mixture. After incubation with the reagent mixture for 60 min (pH 9.5) or 120 min (pH 7.4), the absorbance was measured at 450 nm using the EnVision 2104 Multilabel Plate Reader (PerkinElmer, Waltham, MA) (excitation filter P450). The final concentrations of Arginase-1 and L-arginine in the assay were respectively 1 nM and 5 mM at pH 9.5, and 5 nM and 2.5 mM at pH 7.4.

The reported values were measured in quadruplicate in four independent experiments. Dose-response curves were fitted with a four-parameter logistic regression using XLFit (IDBS, Guildford, UK) to determine IC_50_ values. Graphs for the manuscript were prepared using Prism (GraphPad Software, San Diego, CA). Since the evaluated inhibitors are competitive inhibitors of Arginase-1, inhibition constants (*K*_i_) were determined using the Cheng-Prusoff equation (eq. (1)) (Cheng and Prusoff, 1973), in which [S] is the substrate concentration and $K_{M}$ is the Michaelis constant of the enzyme for its substrate.

Apparent Michaelis-Menten parameters were determined using the colorimetric urea assay at both pH 7.4 and 9.5 by measurement of initial reaction rates in the presence of varying concentrations of L-arginine.

(1) $$K_{i}=\frac{{IC}_{50}}{1+(\left[S\right]/K_{M})}$$

### 4.4 Thermal shift assay

Purified Arginase-1 was diluted to 0.2 mg/mL in 50 mM glycine pH 9.5 or 50 mM Na_2_HPO_4_ pH 7.4. In a 96-well PCR plate (cat no. 652260; Greiner), 10 μL of the enzyme solution was mixed with 5 μL of 200 µM compound dissolved in MQ. Incubation was performed for 45 min at room temperature, followed by the addition of 5 μL 625 times diluted SYPRO Orange (cat no. S6650; Life Technologies, Eugene, OR) in MQ. The final concentrations in the assay were 0.1 mg/mL Arginase-1, 50 μM inhibitor and 2500 times diluted SYPRO Orange. The plate was sealed with Microseal B Adhesive Sealer (cat. no. MSB1001; Bio-Rad, Hercules, CA) and placed in a CFX96 Real-Time Detection System (Bio-Rad). The temperature was increased from 20 to 95 °C in increments of 0.5 °C during which the SYPRO Orange fluorescence was measured. The resulting datasets were first truncated to contain only the data points that lie between the minimal and maximal fluorescence signals. The remaining data were then fitted to the sigmoidal five-parameter equation (Eq. (2)) (Schulz et al., 2013), in which $F_{\min}$ and $F_{\max}$ are respectively the minimum and maximum fluorescent signals of the melting transition, T is the temperature, a is the hill slope and c is the asymmetric factor. The melting temperature was then determined as the point of inflection of the melting curve (Eq. (3)) (Schulz et al., 2013). The reported values were measured in quadruplicate in four independent experiments.

(2) $$F\left(T\right)=F_{\min}+\frac{F_{\max}-F_{min}}{{\left(1+e^{(T^{\text{'}}-T)/a}\right)}^{c}}$$

(3) $$T_{m}\left(inflection\right)=T^{\text{'}}-a*\ln\left(\frac{1}{c}\right)$$

### 4.5 Surface plasmon resonance

Binding kinetics of the inhibitors were determined by SPR using a Biacore T200 (GE Healthcare). Arginase-1 was immobilized on a Ni-NTA sensor chip by Ni-mediated affinity capturing and amine-coupling to a level of 4000 or 6000 resonance units (RU) using 60 µg/mL Arginase-1 in running buffer 1 (50 mM glycine, pH 9.5, 150 mM KCl, and 0.01% Tween-20) or running buffer 2 (50 mM Na_2_HPO_4_, pH 7.4, 150 mM KCl, and 0.01% Tween-20). The inhibitors were diluted in running buffer from a stock solution in MQ and were injected in an increasing concentration range of 0.1, 0.316, 1, 3.16 and 10 µM. Single cycle kinetics were used for measuring compound binding with a flow rate of 30 µL/min, an association time of 100 s per injection, and a dissociation time of 1800 s. The compound response was subtracted with both the reference channel response and the blank injection. The Biacore Evaluation software was used to fit the data to the Langmuir 1:1 binding model with a χ^2^ values ranging between 0.0024 and 0.30 RU^2^ for R_max_ values of 3.2 to 32 RU, indicating minimal deviation between the fit and the experimental data. This was confirmed by determination of the reliability of the curve fits as described previously (Willemsen-Seegers et al., 2017). All combinations of the inhibitors and pH conditions were measured in at least two technical replicates to determine the kinetic constants *k*_a_, *k*_d_ and *K*_D_. The target residence time (τ) was calculated from the *k*_d_ value using the formula τ = 1/*k*_d_.

### 4.6 Protein crystallography and X-ray diffraction data collection

Crystals of human Arginase-1 were prepared through hanging drop vapor diffusion at 21 °C. Drops containing 1 µL of enzyme solution (3.6 mg/mL human Arginase-1, 50 mM Bicine, pH 8.5, and 100 µM MnCl_2_) and 1 µL of precipitant solution, consisting of 200 mM MIB buffer (sodium malonate, imidazole and boric acid in a 2:3:3 M ratio), pH 4.0, and 22–24% (w/v) PEG 1500, were equilibrated against a reservoir containing 500 µL precipitant solution. Rod-like hexagonal crystals generally appeared within a few days. One day prior to soaking of the crystals with inhibitor, the crystals were washed with soaking solution (200 mM MMT buffer (DL-malic acid, MES and Tris base at 1:2:2 M ratios), pH 7.0 or 9.0, and 22–24% PEG 1500). This was done to remove boric acid from the Arginase-1 active site, since this component of the MIB buffer can also act as an inhibitor (Baggio et al., 1997). The crystals were then gradually soaked with 15 mM of the inhibitor during thirteen days for ABH and four days for CB-1158. Subsequently, the crystals were cryoprotected with soaking solution containing an additional 30% ethylene glycol prior to flash cooling in liquid nitrogen.

X-ray diffraction data were collected at the European Synchrotron Radiation Facility (Grenoble, France) on the ID30A-1 beamline. Diffraction data were integrated using Mosflm, followed by space group analysis and data reduction using Pointless, Aimless and CTruncate in the CCP4i2 program suite (Winn et al., 2011). The crystals all exhibited hemihedral twinning and belonged to the space group P3. The crystal structure of Arginase-1 with CB-1158 was solved by molecular replacement in Molrep using a previously reported Arginase-1/ABH structure (PDB ID: 2AEB) (Di Costanzo et al., 2005) as a search model, while the crystal structures of the Arginase-1/ABH complexes at pH 7.0 and 9.0 were solved using the crystal structure of Arginase-1 with CB-1158 as a search model. The structures were refined using the Refmac5 program with twin refinement in CCP4i2 (Winn et al., 2011) and by manual fitting in WinCoot (Emsley et al., 2010). To prevent over-refinement, reflections with the same Miller (*hkl*) indices were used to calculate the free R-factor for all three structures. The presence of the inhibitor in the Arginase-1 active site of both monomers was established by calculating initial electron density in absence of the ligand and was further confirmed after refinement by calculating an omit map in CCP4i (Winn et al., 2011). The manganese ions were refined anisotropically. All protein structure images were generated using PyMOL 1.7.4.5 (DeLano, 2012). Data collection and refinement details can be found in Table 4. Superposition and subsequent structural comparison of the Arginase-1 complexes was performed separately for each monomer (*i.e.,* A and B) to exclude the effect of relative monomer position from the tertiary structure analyses. The valency of the sodium ions was determined using the CheckMyMetal web server (Zheng et al., 2017). Estimation of p*K*_a_ values was performed in MarvinSketch. The reported B-factors are a measure of the displacement of atoms from their average position. These B-factors represent both static disorder (*i.e.*, the presence of different conformations in different parts of the crystal) and dynamic disorder (*i.e.*, thermal vibration of the atoms), as well as crystal packing artefacts (Rhodes, 1993).

### 4.7 Accession numbers

The crystal structures of the Arginase-1/inhibitor complexes have been deposited in the PDB under IDs 6Q92 (ABH at pH 7.0), 6Q9P (ABH at pH 9.0) and 6QAF (CB-1158 at pH 9.0).

## CRediT authorship contribution statement

**Yvonne Grobben:** Conceptualization, Investigation, Formal analysis, Methodology, Writing - original draft, Visualization. **Joost C.M. Uitdehaag:** Conceptualization, Formal analysis, Methodology, Writing - review & editing. **Nicole Willemsen-Seegers:** Investigation. **Werner W.A. Tabak:** Resources. **Jos de Man:** Resources. **Rogier C. Buijsman:** Conceptualization, Resources. **Guido J.R. Zaman:** Conceptualization, Resources, Supervision, Writing - review & editing.

## Declaration of Competing Interest

The authors declare that they have no known competing financial interests or personal relationships that could have appeared to influence the work reported in this paper.
