# Supplementary material for: Structural insights into human Arginase-1 pH dependence and its inhibition by the small molecule inhibitor CB-1158
Source: J Struct Biol X. 2019 Nov 26;4:100014. doi: 10.1016/j.yjsbx.2019.100014 (PMC7337048; doi:10.1016/j.yjsbx.2019.100014)
Supplement: Supplementary data 1 [file mmc1.pdf]

(a)

```
              N-terminal His-tag
-19       -10       |        10         20         30         40         50
GSSHHHHHH SSGLVPRGSH MSAKSRTIGI IGAPFSKGQP RGGVEEGPTV LRKAGLLEKL KEQECDVKDY

        60         70         80         90        100        110        120
GDLPFADIPN DSPFQIVKNP RSVGKASEQL AGKVAEVKKN GRISLVLGGD HSLAIGSISG HARVHPDLGV

       130        140        150        160        170        180        190
IWVDAHTDIN TPLTTTSGNL HGQPVSFLLK ELKGKIPDVP GFSWVTPCIS AKDIVYIGLR DVDPGEHYIL

       200        210        220        230        240        250        260
KTLGIKYFSM TEVDRLGIGK VMEETLSYLL GRKKRPIHLS FDVDGLDPSF TPATGTPVVG GLTYREGLYI

       270        280        290        300        310        320
TEEIYKTGLL SGLDIMEVNP SLGKTPEEVT RTVNTAVAIT LACFGLAREG NHKPIDYLNP PK
```

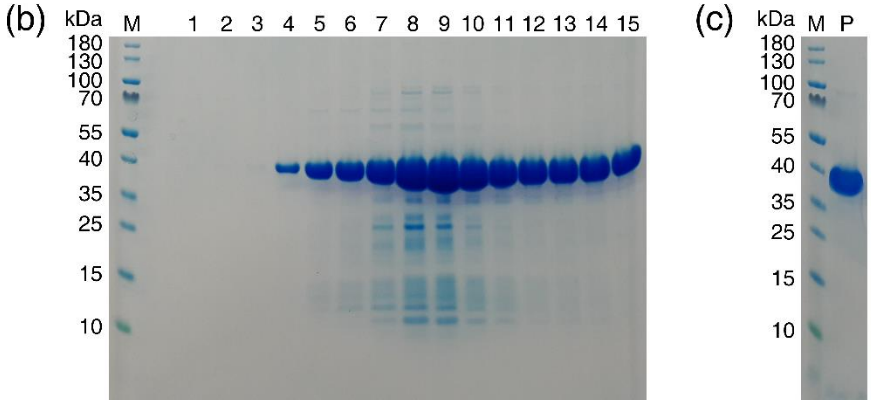

Figure S1. Amino acid sequence and SDS-PAGE analysis of the recombinantly expressed human Arginase-1 enzyme. (a) Amino acid sequence of full-length human Arginase-1 containing an N-terminal hexa-histidine tag and thrombin-cleavable linker. The N-terminal tag is indicated with negative numbers to allow direct comparison of this sequence with other human Arginase-1 sequences. (b) SDS-PAGE analysis of the fractions obtained during affinity chromatography (indicated by the numbers 1 to 15). Fractions 6 to 15 were combined to obtain the purified enzyme preparation shown in panel (c). M: marker; P: purified enzyme preparation.

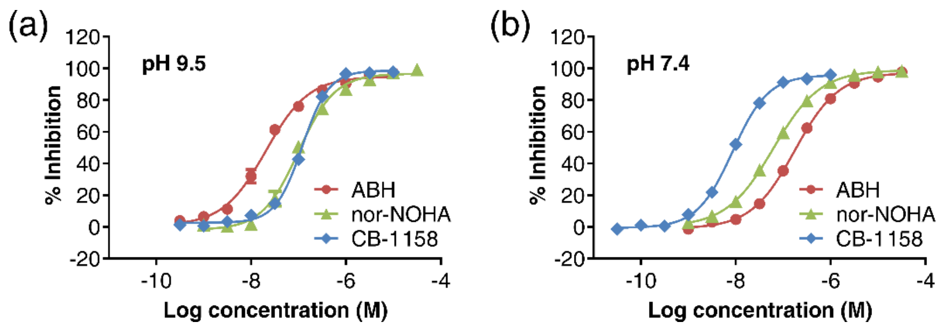

Figure S2. Dose-response curves of inhibitors in the Arginase-1 colorimetric urea assay. (a) Dose-response curves of ABH, nor-NOHA and CB-1158 at pH 9.5 and (b) at pH 7.4. The data points represent the mean of four technical replicates. Error bars indicate standard deviation. Missing error bars indicate that these errors are smaller than the symbol size.

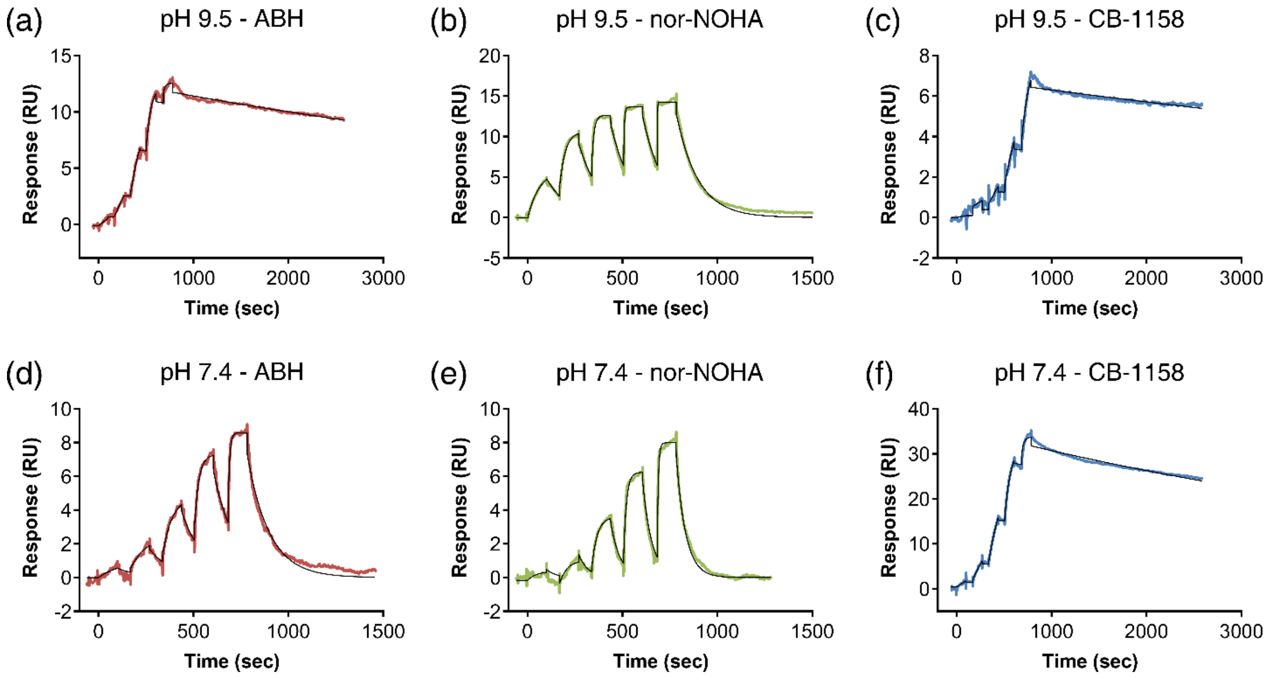

Figure S3. Individual surface plasmon resonance sensorgrams of Arginase-1 inhibitors at pH 9.5 and 7.4. The displayed graphs present the same data sets as in Figure 3, but with the absolute responses and a longer time scale for the inhibitors with long target residence times. The colored lines show the actual response determined by SPR, while the black lines displays the fit obtained using a 1:1 binding model.
